# Dr. Frank C. Davie, L. I. C. H.

**Published:** 1914-10

**Authors:** 


Dr. Frank C. Davie, L. I. C. H., 1876, died at his home in Angelica, N. Y., May 26.

